# Antiviral Agents in Development for Zika Virus Infections

**DOI:** 10.3390/ph12030101

**Published:** 2019-06-29

**Authors:** Mariana Baz, Guy Boivin

**Affiliations:** Research Center in Infectious Diseases of the CHU of Québec and Université Laval, Québec City, QC G1V 4G2, Canada

**Keywords:** Zika virus, antiviral agents, small molecules, virus targets, host targets, repurposing, pre-clinical studies

## Abstract

In 1947, Zika virus (ZIKV), a mosquito-borne flavivirus was identified in Uganda and subsequently spread to Asia and the Pacific regions. In 2015, it was introduced in Brazil causing an important social and sanitary alarm due to its increased virulence and rapid dissemination. Importantly, ZIKV infections have been associated with severe neurological complications such as Guillain–Barré syndrome and microcephaly in fetuses and newborns. Although enormous efforts were made by investigators in the development of effective countermeasures against ZIKV, there is still no approved specific antiviral drug for the treatment of ZIKV infections. Herein, we review several anti ZIKV candidates including drugs targeting both the virus (structural proteins and enzymes) and cellular elements.

## 1. Introduction

Zika virus (ZIKV) is a member of the *Flaviviridae* family and *Flavivirus* genus [1]. The flavivirus genus is the largest among the *Flaviviridae* family with 53 different species [2] and can be further divided into non-vector, tick-borne, and mosquito-borne clusters [1]. Mosquito-borne flaviviruses such as ZIKV, dengue virus (DENV), West Nile virus (WNV), yellow fever virus (YFV), and Japanese encephalitis virus (JEV), have become an increasing public health concern over the last decade since their global incidence has grown dramatically. 

ZIKV (strain MR 766) was first isolated in 1947 [3] from serum samples of a Rhesus monkey during investigation on YFV in the Zika forest of Uganda. In 1948, the virus was isolated from a pool of *Aedes Africanus* (*Stegomyia*) mosquitoes in the same forest [4]. The first human cases of ZIKV infections were reported in Africa in the 1950s [5] and later in Asia but remained restricted to these regions until 2007, when a large outbreak occurred in Yap Island, Federated States of Micronesia followed by outbreaks in French Polynesia, New Caledonia, and the Cook Islands in 2013 and 2014 [6]. In May 2015, ZIKV spread across the Pacific Ocean and was introduced in Brazil where it caused more than one million cases [7]. As of May 2019, the virus rapidly spread to 84 countries, territories or subnational areas, and became a public health problem worldwide [8]. 

ZIKV infection is usually asymptomatic or produces a relatively mild illness with an uneventful recovery [6]. However, during the 2015 epidemics in South America, ZIKV became a global health threat, showing increased virulence, rapid spread, and an association with severe neurological complications such as an unexpected rise of microcephaly cases in fetuses and newborns and a remarkable increase in Guillain–Barré syndrome cases [8,9]. ZIKV is transmitted to people primarily through the bite of infected female *Aedes* species mosquitoes (i.e., *Aedes aegypti* and *Aedes albopictus*) [10,11]. ZIKVs can infect different tissues including reproductive tissues and organs [12,13,14]. In males, ZIKV can infect testes, the prostate, and seminal vesicles [14,15], whereas in females it can infect the vagina, uterus, vaginal epithelium, uterine fibroblasts, trophoblasts, and endothelial cells from the placenta [14,16]. Therefore, direct human–to–human transmission of ZIKV can occur perinatally [17], sexually [18,19], and through breastfeeding [20,21] or blood transfusion [22,23]. 

To date, there is no vaccine or drug licensed to prevent or treat ZIKV infections. The aim of this review article is to provide an update on the search for anti-ZIKV agents. 

## 2. Genome and Replicative Cycle

**Figure 1:**
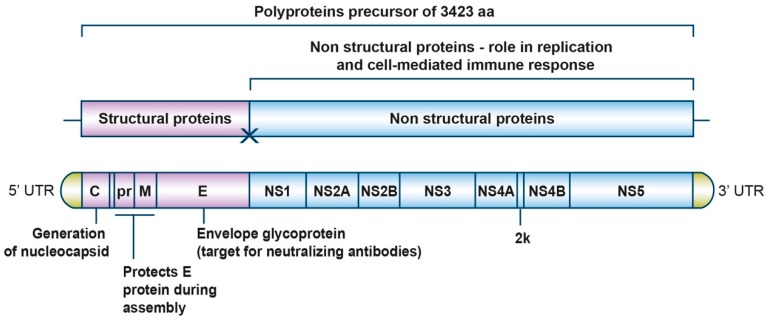
Detailed structure of Zika virus (ZIKV) genome. ZIKV genomic RNA is capped but lacks poly A tail. The viral RNA codes for a polyprotein that is co-translationally cleaved to yield 11 proteins: three structural proteins (C prM/M and E) and eight non-structural proteins (NS1, NS2A, NS2B, NS3, NS4A, 2K, NS4B, and NS5).

The genome of ZIKV (strain MR 766) comprises a 10.8-kb single-stranded positive-sense RNA molecule that contains approximately a 100-nucleotide 5’ untranslated region (UTR), a single open reading frame (ORF) of 10 kb, and approximately 420 nucleotides in the 3’ UTR (Figure 1). The ORF codes for a large polyprotein precursor of 3423 amino acids that is later co- and post-translationally cleaved into three structural (C, prM, and E) and eight nonstructural (NS1, NS2A, NS2B, NS3, S4A, 2K, NS4B, and NS5) proteins (Figure 1 and Table 1). 

The structural proteins are responsible for forming the virus particle and are involved in virus entry, assembly, and release of new virions into the host cell. The capsid protein binds the genomic RNA to form the nucleocapsid core, and the E and prM glycoproteins are viral surface proteins attached to the host-derived lipid envelope. 

The non-structural proteins form the viral replication complex inside the host cell [24]. NS3 and NS5 proteins have enzymatic activities. NS3 encodes serine protease [25,26], RNA helicase [27], nucleoside triphosphatase (NTPase) [28], and RNA triphosphatase (RTPase) [29] enzymatic activities. NS3 is well conserved among the Flaviviruses, with amino acid sequence identity of about 65% between ZIKV, WNV, JEV, and YFV. The NS3 protein contains two domains, an N-terminal protease domain and a C-terminal helicase domain, which are connected via a short linker. Due to the multiple roles played by the NS3 protein in virus life cycle, this protein is an attractive target for antiviral drug discovery. NS5 encodes methyl- and guanylyltransferase (MTase and GTase) enzymatic activities as well as an RNA-dependent RNA polymerase (RdRp) [30]. NS2A, NS2B, NS4A, 2K, and NS4B are transmembrane proteins located within the endoplasmic reticulum (ER) membrane [31]. Some regions of NS2B, NS4A, and NS4B also interact with NS3 and thus anchor the replication complex formed by NS3 and NS5 to the ER membrane [32]. Additionally, NS2B serves as an essential cofactor for the NS3 protease and NS4B blocks interferon α/β signaling [33]. NS4A induces ER membrane rearrangements that are involved in forming the viral replication compartments [34] while NS1 is thought to be involved in genome replication by associating with the luminal side of replication compartment and immune evasion through modulation of host defense mechanism [35]. 

**Figure 2:**
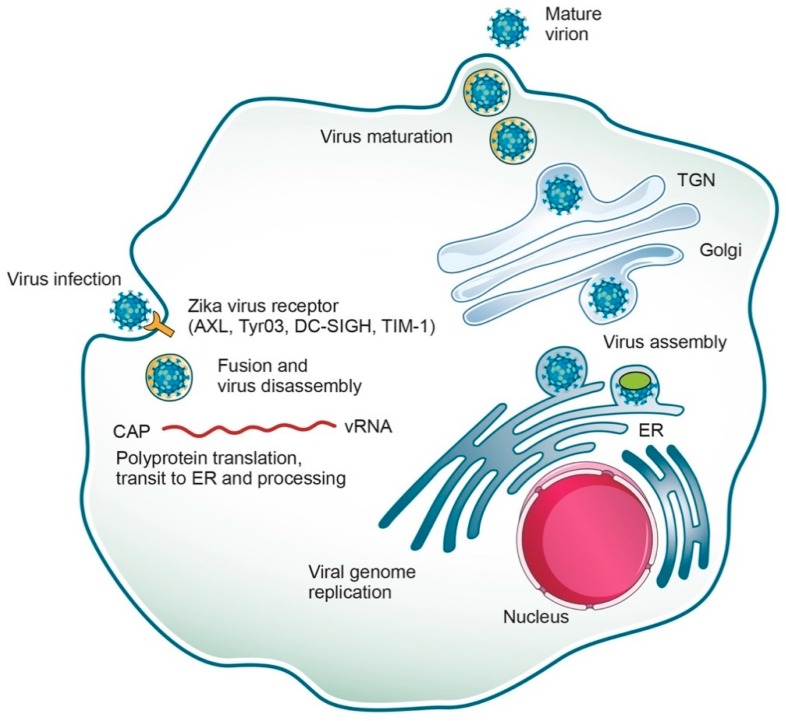
Zika virus life cycle. ZIKV, an enveloped RNA virus, enters cells by receptor-mediated endocytosis and fuses its membranes by an acidic-pH-triggered mechanism in the endosome (via cell surface receptors such as AXL, Tyro3, DC-SIGN and TIM-1) to release the viral RNA. The ssRNA is then translated, and the resulting polyprotein is further cleaved into various structural and non-structural proteins (C, prM, E and NS proteins). Replication takes place in the endoplasmic reticulum (ER) surface. Transcription and replication of the dsDNA results in the formation of new viral mRNA and ssRNA, respectively. Virus assembly takes place in the ER membrane and leads to the formation of immature virions, which are transported through the exocytic pathway. The prM protein is cleaved and the virus is rendered mature. Finally, the mature virus exits the cell via exocytosis.

The replicative cycle of ZIKV is similar to other known flaviviruses (Figure 2). Briefly, the E proteins are involved in the attachment of the virus to receptors on the host membrane, then the virus is internalized via endocytosis [36] mediated by a clathrin protein in a low pH environment. Several cell surface receptors, including the tyrosine-protein kinase receptor AXL, Tyro3, DC-SIGN, and TIM-1, facilitate ZIKV viral entry [37]. The viral genome is released into the host cytoplasm where it is translated, and the resulting polyprotein is proteolytically cleaved into various proteins, the structural and non-structural proteins (C, prM, E and NS proteins) as mentioned above [38,39]. The newly-synthetized (+) RNAs can be either recruited for further rounds of translation/replication or incorporated into the virions, which also initiates their assembly on ER membranes. Assembled virions enter and then travel through the secretory pathway, undergoing maturation along the way, until their final release into the extracellular space. 

## 3. Potential Therapeutic Options for the Treatment of ZIKV Infection

So far, no specific antiviral is approved for the treatment of ZIKV infections. Acetaminophen is usually used to control fever and pain, anti-histamines are used for pruritic rashes, and fluids to prevent dehydration in ZIKV-infected patients. However, acetylsalicylic acid and non-steroidal anti-inflammatory drugs (NSAIDs) are contraindicated because of an increased risk of hemorrhagic syndrome reported with other flaviviral infections as well as the risk of Reye’s syndrome after viral infection in children and teenagers [11]. 

The current search for ZIKV antiviral options is being conducted through several different approaches which target different steps of the replication cycle: by screening of different compound libraries or by the repurposing of drugs with known active efficacy against other diseases and that are already being used in clinic. In addition, natural products [40,41] as well as antibody-based candidates [42,43] are being evaluated but are outside the scope of this review. Antivirals can be classified according to their mode of action in (i) direct-acting antivirals, directed against viral targets, or (ii) host-targeting antivirals, aimed at targeting cellular components needed for the viral cycle. Therefore, in the last few years, a large number of drugs and therapeutic candidates have been discovered or repurposed with ZIKV activity both in vitro and in pre-clinical studies and a few of them have been evaluated in clinical trials (Table 2).

### 3.1. Direct-Acting Antivirals

Virus-directed drugs are those acting against the viral RNA-dependent RNA polymerase (RdRp) (NS5) catalytic domain, including nucleoside analogs and polymerase inhibitors. They target the methyltransferase catalytic domain of the NS5, responsible for transferring the mRNA cap; the NS2B-NS3 trypsin-like serine protease needed for proper processing of the viral polyprotein; and the NS3 helicase.

Nucleoside analogs/derivates, which target viral (not cellular polymerases) to terminate viral RNA replication after incorporation into the viral nascent RNA chain, are safe for use in humans [44], because virus-encoded gene products required for RNA replication can significantly differ in substrate specificity from RNA polymerases involved in host cell replication. Consequently, they have been extensively evaluated against ZIKV in cell culture and in animal models. Eyer et al. tested 29 nucleoside analogues at a concentration of 50 µM for their ability to inhibit cytopathic effects (CPE) of ZIKV on Vero cells. They found that five of these compounds (7-deaza-2’-C-methyladenosine (7-deaza-2-CMA), 2’-C-methyladenosine (2-CMA), 2’-C-methylcythydine (2-CMC), 2’-C-methylguanisine (2-CMG), and 2’-C-methyluridine (2-CMU), significantly reduced cell death compared to non-treated ZIKV infected cells with 50% effective concentration (EC_50_) values ranging from 5.3 to 45.5 µM [45]. A similar observation was found when testing the effects of different 2’-C-methylated nucleosides on the in vitro activity of purified recombinant ZIKV RdRp [46]. In addition, the viral polymerase inhibitor 7-deaza-2-CMA compound exhibited anti-ZIKV activity in Vero cells (EC_50_ = 9.6 µM), decreasing viremia and delaying morbidity and mortality of ZIKV-infected AG129 (interferon (IFN)-α/β and IFN-γ receptor knockout) mice treated once a day with 50 mg/kg/day of the drug [47]. Although 7-deaza-2-CMA inhibitor failed in human clinical trials for chronic hepatitis C treatment, probably due to mitochondrial toxicity [48], this compound could still be suitable and safe for short-term therapy of acute flaviviral diseases, including WNV [49], and thus represents one of the most promising candidates for the treatment of flaviviral infections to date. Favipiravir (6-fluoro-3-hydroxy-2-pyrazinecarboxamide), originally known as T-705, is a novel antiviral compound that selectively and potently inhibits the RdRp of flavi-, orthomyxo-, alpha-, filo-, bunya-, arena-, calici-, noro-viruses, and other RNA viruses [50]. Although the exact mechanism of the interaction of favipiravir with the RdRp molecule has not been fully elucidated, it is hypothesized that favipiravir may be misincorporated in a nascent viral RNA, or it may act by binding to conserved polymerase domains, thus preventing incorporation of nucleotides for viral RNA replication and transcription. Our group and others have demonstrated the ability of favipiravir to inhibit several geographically and temporally different ZIKV strains in in vitro assays [51,52]. The genetic barrier of resistance against favipiravir has been shown to be high for several viruses. In clinical trials of healthy volunteers and in influenza virus-infected patients, favipiravir has been well tolerated. However, caution is needed because of the teratogenic risks of this molecule [53]. 

A pyrimidine synthesis inhibitor such as NITD008 exhibited antiviral activity against ZIKV in vitro (EC_50_ = 0.28–0.95 µM) and in A129 mice treated with 50 mg/kg/day of the drug. Also, 50% of NITD008-treated mice survived without developing any neurological signs [54]. However, due to toxic effects in pre-clinical evaluation, this drug was dismissed [55]. 

The prodrug sofosbuvir is a nucleoside analog that is a RdRp inhibitor commercially available for the treatment of chronic HCV infection. The sofosbuvir phosphoramidate prodrug is converted to its triphosphate analog in the cellular environment to become active. The active metabolite, 2’-fluoro-2-C-methyl-UTP, binds to the active site of NS5 [56] and was shown to inhibit ZIKV infection and replication in human hepatocellular carcinoma (Huh-7) cells, human placental choriocarcinoma (Jar) cells, and SH-Sy5y neuroblastoma cells with EC_50_ values in the range of 0.4–5 μM as well as in human fetal-derived hindbrain and cerebral cortex neuronal stem cells (NSCs) with EC_50_ of about 32 μM [56,57,58]. However, it did not exhibit anti-ZIKV inhibitory activity in Vero cells. Therefore, the inhibitory activity of sofosbuvir varied among different cell types [58]. Interestingly, sequence analysis of ZIKV isolated from infected cells treated with sofosbuvir showed higher frequency of mutations compared with untreated cells [58] suggesting that, besides its inhibitory effect, the drug also increases the incorporation of mutations in the viral genome, increasing error-prone replication [59]. In wild-type (WT) C57BL/6 mice treated with an anti-IFN-α receptor 1 (IFN-αR1) blocking antibody [60], therapeutic oral administration of sofosbuvir with a physiologically relevant dose of 33 mg/kg/day for seven days, protected the animals against ZIKV-induced weight loss and death in 50% of the treated mice. However, higher concentrations of the drug were not effective and resulted in toxicity [57]. Importantly, in clinical phase II and III studies, sofosbuvir was found to be clinically safe and efficacious in patients treated for HCV infection [61]. Sofosbuvir is a class B drug and can be used in men and non-pregnant women to prevent tissue damage. 

BCX4430, an adenosine nucleoside analog, is a selective inhibitor of the viral RdRp. This compound has already been shown to have broad-spectrum activity against a wide range of RNA viruses including WNV, YFV, Marburg and Ebola viruses [62,63]. BCX4430 acts on NS5 polymerase, promoting chain termination of viral RNA synthesis [62]. It was found to inhibit ZIKV replication in Vero cells with EC_50_ values in the range of 3.8–11.7 µg/ml with selective index values of 5.5 and 11.6 depending on the viral strain [64]. In addition, treatment of AG129 mice infected with ZIKV (Malaysian strain, P-6-740) twice daily (BID) with an intramuscular dose of 300 mg/kg of BCX4430 significantly reduced viremia and protected 87.5% of treated mice from mortality. This protection was also observed when treatment was initiated 24 h after infection [64]. This compound is currently in Phase 1 clinical trials to evaluate its safety, tolerability and pharmacokinetics in 94 healthy subjects aged 18–50 years. The results of this study have not been disclosed yet. 

The methyltransferase catalytic domain of the NS5 is responsible for capping the 5’ end of viral genomic RNA. The metal binding pocket of RdRp and SAH/SAM (S-adenosyl-L-homocysteine/S-adenosyl-L-methionine) binding pocket of MTase are classically used in drug screening. Our group has performed virtual screening with a library of 28,341 compounds using a hydrophobic site close to the SAM pocket and identified 10 candidates showing decisive contacts with the MTase. Plaque reduction assay revealed EC_50_ values in the range of 4.8–17.6 µM. However, their in vivo efficacy has not yet been evaluated [65]. Hercik et al. reported the crystal structure of the ZIKV methyltransferase in complex with the pan-methyltransferase inhibitor sinefungin, an adenosine derivative, originally isolated from *Streptomyces griseoleus* as a potential antifungal drug [66]. This compound competes with SAM, the natural substrate of numerous MTases [67]. Sinefungin attaches to GTP and GDP analogs and might be useful in enhancing their affinity toward the enzyme for better selectivity and inhibition of ZIKV replication [68]. However, when this drug was used as anti-parasitic agent in studies conducted in dogs and goats, it was toxic, which has hampered its clinical use [69]. 

NS2B-NS3 trypsin-like serine protease plays a key role in virus replication by contributing to viral polyprotein processing. Studies done by Lee et al. identified 10 compounds with inhibitory activity (EC_50_ < 50 µM) and binding activity (K_D_ of ∼5–10 μM) against the Zika NS2B-NS3 protease from testing 71 HCV NS3/NS4A inhibitors that were initially discovered by high-throughput screening of ∼40,000 compounds [70]. Many natural products such as polyphenols, which have antiviral activity against different viruses (influenza virus, DENV, coronaviruses, HIV-1, hepatitis B virus, etc.) [71,72,73], have been tested against NS2B-NS3 protease, and some of them have been found to inhibit ZIKV protease activity. Lim et al. evaluated 22 polyphenol compounds and found that seven had an EC_50_ ranging from 22 to 113 μM [74]. Roy et al. identified five flavonoids (myricetin, quercetin, luteolin, isorhamnetin, apigenin) and one natural phenol (curcumin) which were shown to inhibit Zika NS2B-NS3 protease by binding to a pocket on the back of the active site and allosterically affect the structure-activity property of Zika NS2B-NS3 protease. The EC_50_ from the flavonoids ranged between 1.3 and 56.3 μM whereas the curcumin EC_50_ was 3.5 μM [75]. Another group screened a total of 2816 Food and Drug Administration (FDA)-approved drugs and investigational drugs and found that 23 compounds had EC_50_ below 15 μM. However, 12 of those compounds were considered Pan-Assay Interference Compounds (PAINS). Three (temoporfin, niclosamide, and nitazoxanide) of the 12 remaining compounds had an EC_50_ value ranging from 1.1 to 15.9 μM. Temoporfin displayed a very low EC_50_ value (nanomolar range) and, when tested in a lethal mouse model, was able to inhibit viremia and protect 83% of infected mice. In addition, mice that survived did not present any signs of neurological disorder [76]. Similarly, a study done by Yuan et al. using an in-silico structure-based approach to rapidly screen a large chemical library of 8277 compounds, successfully identified eight clinically approved drugs with inhibitory activity on the ZIKV NS2B-NS3 protease [77]. In addition, the authors further validated the anti-ZIKV activity of novobiocin, an aminocoumarin antibiotic, using in vitro antiviral assays and in an immunodeficient mouse model. In vitro, novobiocin had an EC_50_ value of 24.82 μM and treatment of mice with 100 mg/kg of the drug BID from day 1 to 13 post-infection, significantly (*p* < 0.05) increased survival rate (100% vs. 0%), decreased mean blood and tissue viral loads, and produced less severe histopathological changes than untreated controls [77]. 

NS3 helicases display adenosine triphosphatase (ATPase) and RNA triphosphatase (RTPase) activities. NS3 inhibitors can be used to impede ZIKV infection. Suramin, an anti-parasitic drug used to treat trypanosomal human sleeping sickness, is available for prophylactic and therapeutic use in children. This drug was also shown to inhibit multiple DNA and RNA viruses including DENV, herpes simplex virus type 1, cytomegaloviruses human hepatitis B, hepatitis D, hepatitis C, bunyaviruses, enterovirus 71, and others [78,79,80,81]. Suramin was also able to inhibit enterovirus 71 by neutralizing virus particles prior to attachment and chikungunya virus in mice [82,83,84]. Albulescu et al. showed that suramin has anti-ZIKV activity, with an EC_50_ of 39.8 μM, by interfering with viral attachment and the release of infectious progeny from ZIKV-infected cells [85]. When treatment was initiated post-entry, viral RNA synthesis was unaffected but both the release of genomes and the infectivity of ZIKV were reduced, suggesting that this drug also affects virus biogenesis probably by interfering with glycosylation and maturation of ZIKV during traffic through the secretory pathway [85]. 

### 3.2. Host-Targeting Antivirals

Targeting host cell processes provides an attractive broad-spectrum strategy because they are often employed by multiple viruses and, in addition, they are less prone to develop drug resistance [86]. These host-acting inhibitors can be directed to any molecule or pathway implicated in the different steps of the viral life cycle, from binding, entry and fusion, to the formation of the replication complex, viral maturation, and egress. 

In order to maintain proper replication, viruses rely on the supply of nucleosides from the host cells. Ribavirin is a guanosine analogue that has broad-spectrum activity against several RNA and DNA viruses [87,88]. Different mechanisms have been proposed to explain ribavirin’s antiviral properties including indirect mechanisms such as inosine monophosphate dehydrogenase inhibition (IMPDH) and immunomodulatory effects as well as direct mechanisms such as interference with RNA capping, polymerase inhibition, and lethal mutagenesis [87]. This antiviral is usually employed in combination therapies to treat chronic HCV infections. Our group and others demonstrated the inhibitory activity of ribavirin against ZIKV strains of different geographic origins in several cell lines such as Vero cells, human neural progenitor cells (hNPCs), human dermal fibroblasts (HDFs), and human lung adenocarcinoma cells (A549) [51,89]. Ribavirin was also shown to suppress viremia in ZIKV-infected STAT-1-deficient mice, which lack type I IFN signaling and are thus highly sensitive to ZIKV infection with a lethal outcome [89]. Recent studies have shown that merimepodib (MMPD or VX-497) and mycophenolic acid (MPA), two IMPDH inhibitors, also inhibit ZIKV-RNA replication in different cell types, including Huh-7 cells, human cervical placental cells, and neural stem and primary amniotic cells [90,91,92]. Azathioprine, another inhibitor of the purine synthesis and immunosuppressive compound, was shown to abolish ZIKV replication in HeLa (cervical cancer cells) and JEG3 (human choriocarcinoma cell line) cells; however, its use in pregnant women is not recommended [92].

Similar to the purine synthesis inhibitors, compounds inhibiting the synthesis of pyrimidines have also been shown to affect ZIKV replication. Pascoalino et al. screened a library of 725 compounds from a collection of chemically diverse FDA-approved drugs with known and unknown mechanisms of action. The entire library was screened at 20 µM against ZIKV infecting Huh7 cells. The authors identified the 6-azauridine (EC_50_ = 2.3 μM) and another pyrimidine biosynthesis inhibitor, 5-fluorouracil (EC_50_ = 14.3 μM), which inhibit thymidylate synthase (the enzyme that catalyzes the final step of thymidine biosynthesis) [93]. These compounds are classified in pregnancy category D by the FDA [92] and thus have human fetal risk which is not unexpected because they deplete the cellular pool of nucleotides, affecting proper development of the fetus. In addition, the authors have identified lovastatin, a 3-hydroxy-3-methylglutaryl-coenzyme (HMG-CoA) reductase inhibitor, whose activity against ZIKV was confirmed through a dose-response assay (EC_50_ = 20.7 μM) [93]. The antiflaviviral activity of lovastatin was also demonstrated against HCV and DENV [94,95]. In addition, Sarkey et al., demonstrated that a short-term parenteral course of high-dose lovastatin in mice markedly attenuated nervous system injury and, thus, could be eventually used in inflammatory peripheral nerve diseases such as Guillain–Barré syndrome (GBS), a consequence of ZIKV infection [96]. 

Retallack et al. performed the screening of 2177 clinically approved compounds by monitoring inhibition of virus-dependent cell death at 72 h post-infection (hpi) in Vero cells. The screening identified several compounds that rescued cell viability, including antibiotics and inhibitors of nucleotide and protein synthesis with many that showed toxicity in Vero or U87 cells or were contraindicated during pregnancy. However, they identified the macrolide antibiotic azithromycin, which rescued ZIKV-induced cytopathic effect in glial cells with low toxicity and reduced ZIKV infection of U87 cells at an EC_50_ of 2 to 3 µM [97]. This drug is generally safe during pregnancy [98] and was suggested as a potential option to prevent GBS and microcephaly [97].

Chloroquine, an anti-inflammatory FDA-approved 4-aminoquinoline, is an old antimalarial drug which can be prescribed to pregnant women at risk of exposure to *Plasmodium* parasites [99,100]. This drug has shown antiviral activity against several viruses, through the inhibition of pH-dependent steps of viral replication, including anti-ZIKV activity in Vero cells, human brain microvascular endothelial cells (hBMECs), and human neural stem cells (NSCs) with EC_50_ values of 9.82, 14.2, and 12.36 μM, respectively. In addition, chloroquine was able to partially reverse morphological changes induced by ZIKV in mouse neurospheres [101]. In vitro, chloroquine reduces the number of ZIKV-infected cells, and inhibits virus production and cell death promoted by ZIKV infection without cytotoxic effects [101]. 

Saliphenylhalamide (SaliPhe), a viral entry blocker which targets vacuolar ATPase and prevents the acidification of endosomes, also inhibits ZIKV replication in human retinal pigment epithelial (RPE) cells, which are natural targets for ZIKV infection [102] with an EC_50_ of 1 μM [103]. Similarly, obatoclax mesylate, also known as GX15-070, is an experimental drug for the treatment of different types of cancer. This drug is an inhibitor of the Bcl-2 family of proteins that targets cellular Mcl-1 and inhibits endocytosis, thus inducing apoptosis. Obatoclax mesylate displays an EC_50_ of 0.3 μM against ZIKV. Niclosamide is an FDA-approved drug, formerly designated in pregnancy category B, that has been used for more than 50 years showing acceptable safety. It has been broadly used in the treatment of intestinal helminthiasis. This drug blocks the acidification of endosomes, using a mechanism that has not yet been fully elucidated [104]. In glioblastoma SNB-19 cells, the EC_50_ against ZIKV was 0.37 μM based on the measurement of intracellular viral RNA [105]. PHA-690509, is an investigational cyclin-dependent kinase inhibitor which inhibited ZIKV infection with an EC_50_ value of 1.72 μM [105]. This drug was detected by Xu et al. as part of a large drug repurposing screen for ZIKV. The authors measured ZIKV-induced caspase-3 activity and cell viability from over 6000 approved drugs and drug candidate compounds using human neural cells. This study led to the identification of small molecules that either protect against cell death in multiple neural cell types or inhibit ZIKV replication. Among them, seliciclib (a purine analog) and RGB-286147 inhibited ZIKV infection at sub-micromolar concentrations [105]. 

Studies from Costa et al. showed that ZIKV has tropism for the central nervous system (CNS) and replicates preferentially in neurons, inducing neurodegeneration, neuroinflammation, and ophthalmologic disorders [106]. Neurodegeneration in ZIKV disease possibly occurs due to the excitotoxicity of glutamate. FDA-approved *N*-methyl-D-aspartate receptor (NMDAR) antagonistic drugs to treat Alzheimer disease such as memantine, MK-801, agmatine, and ifenprodil were found to prevent neuronal cell death caused by ZIKV under in vitro conditions without reducing viral titers [106]. Blocking hyperactivation of NMDAR would therefore reduce rates of Zika virus-induced cell death and help ameliorate neuronal symptoms during infection. Costa et al. showed that memantine was very effective at preventing ZIKV-induced neuronal cell death and neurodegeneration in IFN-α/βR^−/−^mice [106]. Importantly, memantine is also listed in pregnancy category B drugs by the FDA. Therefore, it could be used safely to reduce neurological complications associated with ZIKV infection. 

## 4. Who Benefits from ZIKV Therapies?

In immunocompetent individuals, ZIKV infection is asymptomatic or produces a relatively mild illness with an uneventful recovery that can be treated with acetaminophen, anti-histamines, and fluids. However, due to the ability of ZIKV to infect fetuses and cause severe neurological disease, there is a need for anti-zika drugs that function during pregnancy and which are safe for both the pregnant mother and her fetus. Many FDA approved drugs have been tested for efficacy against ZIKV and can be repurposed for treating ZIKV infection in humans. In order to reach the fetus, these drugs must be able to cross the placental barrier and the blood–brain barrier to reach neural cells, the main targets of ZIKV. So far, however, no drug has been identified that is clinically safe for use in both pregnant women and fetus. The World Health Organization (WHO) recommends that niclosamide may be used during pregnancy as an anthelminthic agent (with anti-ZIKV activity) because it has not been shown to be mutagenic, teratogenic or embryotoxic (http://apps.who.int/medicinedocs/en/d/Jh2922e/3.1.3.html#Jh2922e.3.1.3). The US Centers for Disease Control and Prevention (CDC) further recommend that “for individual patients in clinical settings, the risk of treatment with niclosamide in pregnant women who are known to have an infection needs to be balanced with the risk of disease progression in the absence of treatment” (http://www.cdc.gov/parasites/hymenolepis/health_professionals/). In addition, niclosamide and other direct- or indirect-acting agents could be used to reduce viral load in infected men and nonpregnant women (potentially those at higher risk due to immunosuppression), reducing transmission and potentially preventing Guillain–Barré syndrome and other ZIKV-related neurological complications in humans. 

## 5. Conclusions

In the last few years, researchers have invested efforts in the development of a vaccine and antiviral drugs for the prevention or treatment of ZIKV infections. However, there is still no approved vaccine or drug available for this emerging pathogen. Different approaches and methodologies have been used, from testing specific drugs with known antiviral activity for other viruses, to testing libraries composed of hundreds or thousands of bioactive compounds that have already gone through several steps of drug approval by regulatory agencies (repurposing drugs). Most antiviral candidates have been evaluated in vitro and some of them have also been tested in animal models. However, few candidates have advanced into clinical trials. Further development of novel compounds as well as combination therapies may open new avenues for the treatment of ZIKV-related diseases. 

## Figures and Tables

**Table 1:** The table shows the structural and nonstructural ZIKV proteins from the 5’ to the 3’ untranslated regions. The name, size in amino acids, and function are shown for each protein.

Name	Amino Acid Residues	Function
C	122	Generation of nucleocapsid (encapsulates genomic RNA)
prM	168	Protects E protein during assembly
E	500	Envelope glycoprotein (membrane binding and fusion)
NS1	352	Replication and immune response regulation
NS2A	226	Replication and capsid assembly
NS2B	130	NS3 cofactor
NS3	617	Serine protease NTPase, RNA helicase
NS4A	127	Viral membrane formation
2K	23	Signal peptide
NS4B	251	Inhibits antiviral state
NS5	903	RNA dependent RNA polymerase

**Table 2:** Summary of the different antiviral options described in this manuscript.

**Direct-Acting Antivirals**			
**Name**	**Mode of action**	**In vitro**	**In vivo**
7-deaza-2-CMA	RdRp inhibitor	√	√
2-CMA, 2-CMC, 2-CMG, 2-CMU	RdRp inhibitor	√	X
Favipiravir	RdRp inhibitor	√	X
NITD008	Pyrimidine synthesis inhibitor	√	√
Sofosbuvir	RdRp inhibitor	√	√
BCX4430 *	RdRp inhibitor	√	√
Sinefungin	Pan-methyltransferase inhibitor	√	X
Myricetin, quercetin, luteolin, isorhamnetin, apigenin, curcumin	NS2B-NS3 protease inhibitor	√	X
Niclosamide, and nitazoxanide	NS2B-NS3 protease inhibitor	√	X
Temoporfin	NS2B-NS3 protease inhibitor	√	√
Novobiocin	NS2B-NS3 protease inhibitor	√	√
Suramin	NS3 inhibitor	√	X
**Host-Targeting Antivirals**			
**Name**	**Mode of action**	**In vitro**	**In vivo**
Ribavirin	Several mechanisms including purine synthesis inhibitor	√	√
Merimepodib and mycophenolic acid	Inosine monophosphate dehydrogenase (IMPDH) inhibitors	√	X
Azathioprine	Purine synthesis inhibitor	√	X
6-azauridine, 5-fluorouracil	Pirimidine synthesis inhibitor	√	X
lovastatin	HMG-CoA reductase inhibitor	√	X
Azithromycin	Unknown mechanisms of action against ZIKV	√	X
Chloroquine	Inhibition of pH-dependent steps of viral replication	√	X
Saliphenylhalamide	Viral entry inhibitor	√	X
Obatoclax mesylate (GX15-070)	Bcl-2 protein inhibitor	√	X
PHA-690509	Cyclin-dependent kinase inhibitor	√	X
MK-801, agmatine, and ifenprodil	Neuronal cell death inhibitor	√	X
Memantine	Neuronal cell death inhibitor	√	√

* Denotes drugs under clinical trials, X: not done. HMG-CoA: 3-hydroxy-3-methylglutaryl-coenzyme.

## References

[1] Kuno G., Chang G.J., Tsuchiya K.R., Karabatsos N., Cropp C.B. (1998). Phylogeny of the genus Flavivirus. J. Virol..

[2] Centers for Disease Control and Prevention (CDC) (2009). Oseltamivir-resistant 2009 pandemic influenza A (H1N1) virus infection in two summer campers receiving prophylaxis-North Carolina, 2009. Morb. Mortal. Wkly. Rep..

[3] Dick G.W. (1952). Zika virus. II. Pathogenicity and physical properties. Trans. R. Soc. Trop. Med. Hyg..

[4] Haddow A.J., Williams M.C., Woodall J.P., Simpson D.I., Goma L.K. (1964). Twelve Isolations of Zika Virus from Aedes (Stegomyia) Africanus (Theobald) Taken in and above a Uganda Forest. Bull. World Health Org..

[5] Simpson D.I. (1964). Zika Virus Infection in Man. Trans. R. Soc. Trop. Med. Hyg..

[6] Duffy M.R., Chen T.H., Hancock W.T., Powers A.M., Kool J.L., Lanciotti R.S., Pretrick M., Marfel M., Holzbauer S., Dubray C. (2009). Zika virus outbreak on Yap Island, Federated States of Micronesia. N. Engl. J. Med..

[7] Zanluca C., Melo V.C., Mosimann A.L., Santos G.I., Santos C.N., Luz K. (2015). First report of autochthonous transmission of Zika virus in Brazil. Mem. Inst. Oswaldo Cruz.

[8] Hamelin M.E., Baz M., Abed Y., Couture C., Joubert P., Beaulieu E., Bellerose N., Plante M., Mallett C., Schumer G. (2010). Oseltamivir-resistant pandemic A/H1N1 virus is as virulent as its wild-type counterpart in mice and ferrets. PLoS Pathog..

[9] Rasmussen S.A., Jamieson D.J., Honein M.A., Petersen L.R. (2016). Zika Virus and Birth Defects—Reviewing the Evidence for Causality. N. Engl. J. Med..

[10] Diagne C.T., Diallo D., Faye O., Ba Y., Faye O., Gaye A., Dia I., Faye O., Weaver S.C., Sall A.A. (2015). Potential of selected Senegalese Aedes spp. mosquitoes (Diptera: Culicidae) to transmit Zika virus. BMC Infect. Dis..

[11] Musso D., Gubler D.J. (2016). Zika Virus. Clin. Microbiol. Rev..

[12] Gourinat A.C., O’Connor O., Calvez E., Goarant C., Dupont-Rouzeyrol M. (2015). Detection of Zika virus in urine. Emerg. Infect. Dis..

[13] Coffey L.L., Pesavento P.A., Keesler R.I., Singapuri A., Watanabe J., Watanabe R., Yee J., Bliss-Moreau E., Cruzen C., Christe K.L. (2017). Zika Virus Tissue and Blood Compartmentalization in Acute Infection of Rhesus Macaques. PLoS ONE.

[14] Miner J.J., Diamond M.S. (2017). Zika Virus Pathogenesis and Tissue Tropism. Cell Host Microbe.

[15] Govero J., Esakky P., Scheaffer S.M., Fernandez E., Drury A., Platt D.J., Gorman M.J., Richner J.M., Caine E.A., Salazar V. (2016). Zika virus infection damages the testes in mice. Nature.

[16] Hirsch A.J., Smith J.L., Haese N.N., Broeckel R.M., Parkins C.J., Kreklywich C., DeFilippis V.R., Denton M., Smith P.P., Messer W.B. (2017). Zika Virus infection of rhesus macaques leads to viral persistence in multiple tissues. PLoS Pathog..

[17] Besnard M., Lastere S., Teissier A., Cao-Lormeau V., Musso D. (2014). Evidence of perinatal transmission of Zika virus, French Polynesia, December 2013 and February 2014. Eurosurveillance.

[18] Imperato P.J. (2016). The Convergence of a Virus, Mosquitoes, and Human Travel in Globalizing the Zika Epidemic. J. Commun. Health.

[19] Foy B.D., Kobylinski K.C., Chilson Foy J.L., Blitvich B.J., Travassos da Rosa A., Haddow A.D., Lanciotti R.S., Tesh R.B. (2011). Probable non-vector-borne transmission of Zika virus, Colorado, USA. Emerg. Infect. Dis..

[20] Dupont-Rouzeyrol M., Biron A., O’Connor O., Huguon E., Descloux E. (2016). Infectious Zika viral particles in breastmilk. Lancet.

[21] Calvet G., Aguiar R.S., Melo A.S.O., Sampaio S.A., de Filippis I., Fabri A., Araujo E.S.M., de Sequeira P.C., de Mendonca M.C.L., de Oliveira L. (2016). Detection and sequencing of Zika virus from amniotic fluid of fetuses with microcephaly in Brazil: A case study. Lancet Infect. Dis..

[22] Musso D., Nhan T., Robin E., Roche C., Bierlaire D., Zisou K., Shan Yan A., Cao-Lormeau V.M., Broult J. (2014). Potential for Zika virus transmission through blood transfusion demonstrated during an outbreak in French Polynesia, November 2013 to February 2014. Eurosurveillance.

[23] Marano G., Pupella S., Vaglio S., Liumbruno G.M., Grazzini G. (2016). Zika virus and the never-ending story of emerging pathogens and transfusion medicine. Blood Transfus..

[24] BioCryst Pharmaceuticals Inc. BioCryst’s Partner Shinogi Receives Approval for Pediatric Use of Peramivir in Japan. http://investor.shareholder.com/biocryst/releasedetail.cfm?releaseid=523371.

[25] Chambers T.J., Weir R.C., Grakoui A., McCourt D.W., Bazan J.F., Fletterick R.J., Rice C.M. (1990). Evidence that the N-terminal domain of nonstructural protein NS3 from yellow fever virus is a serine protease responsible for site-specific cleavages in the viral polyprotein. Proc. Natl. Acad. Sci. USA.

[26] Wengler G., Czaya G., Farber P.M., Hegemann J.H. (1991). In vitro synthesis of West Nile virus proteins indicates that the amino-terminal segment of the NS3 protein contains the active centre of the protease which cleaves the viral polyprotein after multiple basic amino acids. J. Gen. Virol..

[27] Li H., Clum S., You S., Ebner K.E., Padmanabhan R. (1999). The serine protease and RNA-stimulated nucleoside triphosphatase and RNA helicase functional domains of dengue virus type 2 NS3 converge within a region of 20 amino acids. J. Virol..

[28] Takegami T., Sakamuro D., Furukawa T. (1995). Japanese encephalitis virus nonstructural protein NS3 has RNA binding and ATPase activities. Virus Genes.

[29] Bartelma G., Padmanabhan R. (2002). Expression, purification, and characterization of the RNA 5’-triphosphatase activity of dengue virus type 2 nonstructural protein 3. Virology.

[30] Guyatt K.J., Westaway E.G., Khromykh A.A. (2001). Expression and purification of enzymatically active recombinant RNA-dependent RNA polymerase (NS5) of the flavivirus Kunjin. J. Virol. Methods.

[31] Xie X., Gayen S., Kang C., Yuan Z., Shi P.Y. (2013). Membrane topology and function of dengue virus NS2A protein. J. Virol..

[32] Erbel P., Schiering N., D’Arcy A., Renatus M., Kroemer M., Lim S.P., Yin Z., Keller T.H., Vasudevan S.G., Hommel U. (2006). Structural basis for the activation of flaviviral NS3 proteases from dengue and West Nile virus. Nat. Struct. Mol. Biol..

[33] Munoz-Jordan J.L., Sanchez-Burgos G.G., Laurent-Rolle M., Garcia-Sastre A. (2003). Inhibition of interferon signaling by dengue virus. Proc. Natl. Acad. Sci. USA.

[34] Roosendaal J., Westaway E.G., Khromykh A., Mackenzie J.M. (2006). Regulated cleavages at the West Nile virus NS4A-2K-NS4B junctions play a major role in rearranging cytoplasmic membranes and Golgi trafficking of the NS4A protein. J. Virol..

[35] Mackenzie J.M., Jones M.K., Young P.R. (1996). Immunolocalization of the dengue virus nonstructural glycoprotein NS1 suggests a role in viral RNA replication. Virology.

[36] Chambers T.J., Hahn C.S., Galler R., Rice C.M. (1990). Flavivirus genome organization, expression, and replication. Annu. Rev. Microbiol..

[37] Hamel R., Dejarnac O., Wichit S., Ekchariyawat P., Neyret A., Luplertlop N., Perera-Lecoin M., Surasombatpattana P., Talignani L., Thomas F. (2015). Biology of Zika Virus Infection in Human Skin Cells. J. Virol..

[38] Van Hemert F., Berkhout B. (2016). Nucleotide composition of the Zika virus RNA genome and its codon usage. Virol. J..

[39] Lindenbach B.D., Rice C.M. (2003). Molecular biology of flaviviruses. Adv. Virus Res..

[40] Batista M.N., Braga A.C.S., Campos G.R.F., Souza M.M., Matos R.P.A., Lopes T.Z., Candido N.M., Lima M.L.D., Machado F.C., Andrade S.T.Q. (2019). Natural Products Isolated from Oriental Medicinal Herbs Inactivate Zika Virus. Viruses.

[41] Haddad J.G., Koishi A.C., Gaudry A., Nunes Duarte Dos Santos C., Viranaicken W., Despres P., El Kalamouni C. (2019). Doratoxylon apetalum, an Indigenous Medicinal Plant from Mascarene Islands, Is a Potent Inhibitor of Zika and Dengue Virus Infection in Human Cells. Int. J. Mol. Sci..

[42] Tychan Pte Ltd. Safety and Tolerability of an Antibody Against Zika Virus (Tyzivumab) in ZIKV Infected Patients. ClinicalTrials.gov. NCT number: NCT03776695. 2018 Dec. NCT03776695. NCT03776695.

[43] Niu X., Zhao L., Qu L., Yao Z., Zhang F., Yan Q., Zhang S., Liang R., Chen P., Luo J. (2019). Convalescent patient-derived monoclonal antibodies targeting different epitopes of E protein confer protection against Zika virus in a neonatal mouse model. Emerg. Microbes Infect..

[44] De Clercq E., Neyts J. (2009). Antiviral agents acting as DNA or RNA chain terminators. Handb. Exp. Pharmacol..

[45] Eyer L., Nencka R., Huvarova I., Palus M., Joao Alves M., Gould E.A., De Clercq E., Ruzek D. (2016). Nucleoside Inhibitors of Zika Virus. J. Infect. Dis..

[46] Hercik K., Kozak J., Sala M., Dejmek M., Hrebabecky H., Zbornikova E., Smola M., Ruzek D., Nencka R., Boura E. (2017). Adenosine triphosphate analogs can efficiently inhibit the Zika virus RNA-dependent RNA polymerase. Antivir. Res..

[47] Zmurko J., Marques R.E., Schols D., Verbeken E., Kaptein S.J., Neyts J. (2016). The Viral Polymerase Inhibitor 7-Deaza-2’-C-Methyladenosine Is a Potent Inhibitor of In Vitro Zika Virus Replication and Delays Disease Progression in a Robust Mouse Infection Model. PLoS Negl. Trop. Dis..

[48] Arnold J.J., Sharma S.D., Feng J.Y., Ray A.S., Smidansky E.D., Kireeva M.L., Cho A., Perry J., Vela J.E., Park Y. (2012). Sensitivity of mitochondrial transcription and resistance of RNA polymerase II dependent nuclear transcription to antiviral ribonucleosides. PLoS Pathog..

[49] Chen Y.L., Yin Z., Lakshminarayana S.B., Qing M., Schul W., Duraiswamy J., Kondreddi R.R., Goh A., Xu H.Y., Yip A. (2010). Inhibition of dengue virus by an ester prodrug of an adenosine analog. Antimicrob. Agents Chemother..

[50] Furuta Y., Takahashi K., Shiraki K., Sakamoto K., Smee D.F., Barnard D.L., Gowen B.B., Julander J.G., Morrey J.D. (2009). T-705 (favipiravir) and related compounds: Novel broad-spectrum inhibitors of RNA viral infections. Antivir. Res..

[51] Baz M., Goyette N., Griffin B.D., Kobinger G.P., Boivin G. (2017). In vitro susceptibility of geographically and temporally distinct Zika viruses to favipiravir and ribavirin. Antivir. Ther..

[52] Cai L., Sun Y., Song Y., Xu L., Bei Z., Zhang D., Dou Y., Wang H. (2017). Viral polymerase inhibitors T-705 and T-1105 are potential inhibitors of Zika virus replication. Arch. Virol..

[53] Delang L., Abdelnabi R., Neyts J. (2018). Favipiravir as a potential countermeasure against neglected and emerging RNA viruses. Antivir. Res..

[54] Deng Y.Q., Zhang N.N., Li C.F., Tian M., Hao J.N., Xie X.P., Shi P.Y., Qin C.F. (2016). Adenosine Analog NITD008 Is a Potent Inhibitor of Zika Virus. Open Forum Infect. Dis..

[55] Munjal A., Khandia R., Dhama K., Sachan S., Karthik K., Tiwari R., Malik Y.S., Kumar D., Singh R.K., Iqbal H.M.N. (2017). Advances in Developing Therapies to Combat Zika Virus: Current Knowledge and Future Perspectives. Front. Microbiol..

[56] Reznik S.E., Ashby C.R. (2017). Sofosbuvir: An antiviral drug with potential efficacy against Zika infection. Int. J. Infect. Dis..

[57] Bullard-Feibelman K.M., Govero J., Zhu Z., Salazar V., Veselinovic M., Diamond M.S., Geiss B.J. (2017). The FDA-approved drug sofosbuvir inhibits Zika virus infection. Antivir. Res..

[58] Sacramento C.Q., de Melo G.R., de Freitas C.S., Rocha N., Hoelz L.V., Miranda M., Fintelman-Rodrigues N., Marttorelli A., Ferreira A.C., Barbosa-Lima G. (2017). The clinically approved antiviral drug sofosbuvir inhibits Zika virus replication. Sci. Rep..

[59] Perales C., Domingo E. (2016). Antiviral Strategies Based on Lethal Mutagenesis and Error Threshold. Curr. Top. Microbiol. Immunol..

[60] Lazear H.M., Govero J., Smith A.M., Platt D.J., Fernandez E., Miner J.J., Diamond M.S. (2016). A Mouse Model of Zika Virus Pathogenesis. Cell Host Microbe.

[61] Mangia A., Piazzolla V. (2014). Overall efficacy and safety results of sofosbuvir-based therapies in phase II and III studies. Dig. Liver Dis..

[62] Eyer L., Zouharova D., Sirmarova J., Fojtikova M., Stefanik M., Haviernik J., Nencka R., de Clercq E., Ruzek D. (2017). Antiviral activity of the adenosine analogue BCX4430 against West Nile virus and tick-borne flaviviruses. Antivir. Res..

[63] Warren T.K., Wells J., Panchal R.G., Stuthman K.S., Garza N.L., Van Tongeren S.A., Dong L., Retterer C.J., Eaton B.P., Pegoraro G. (2014). Protection against filovirus diseases by a novel broad-spectrum nucleoside analogue BCX4430. Nature.

[64] Julander J.G., Siddharthan V., Evans J., Taylor R., Tolbert K., Apuli C., Stewart J., Collins P., Gebre M., Neilson S. (2017). Efficacy of the broad-spectrum antiviral compound BCX4430 against Zika virus in cell culture and in a mouse model. Antivir. Res..

[65] Stephen P., Baz M., Boivin G., Lin S.X. (2016). Structural Insight into NS5 of Zika Virus Leading to the Discovery of MTase Inhibitors. J. Am. Chem. Soc..

[66] Hamil R.L., Hoehn M.M. (1973). A9145, a new adenine-containing antifungal antibiotic. I. Discovery and isolation. J. Antibiot. (Tokyo).

[67] Zhang J., Zheng Y.G. (2016). SAM/SAH Analogs as Versatile Tools for SAM-Dependent Methyltransferases. ACS Chem. Biol..

[68] Hercik K., Brynda J., Nencka R., Boura E. (2017). Structural basis of Zika virus methyltransferase inhibition by sinefungin. Arch. Virol..

[69] Robert-Gero M., Lawrence F., Lederer E., Hart D.T. (1989). Potential Clinical Use of Sinefungin: Reduction of Toxicity and Enhancement of Activity. Leishmaniasis.

[70] Lee H., Ren J., Nocadello S., Rice A.J., Ojeda I., Light S., Minasov G., Vargas J., Nagarathnam D., Anderson W.F. (2017). Identification of novel small molecule inhibitors against NS2B/NS3 serine protease from Zika virus. Antivir. Res..

[71] Bahramsoltani R., Sodagari H.R., Farzaei M.H., Abdolghaffari A.H., Gooshe M., Rezaei N. (2016). The preventive and therapeutic potential of natural polyphenols on influenza. Expert Rev. Anti Infect. Ther..

[72] Vazquez-Calvo A., Jimenez de Oya N., Martin-Acebes M.A., Garcia-Moruno E., Saiz J.C. (2017). Antiviral Properties of the Natural Polyphenols Delphinidin and Epigallocatechin Gallate against the Flaviviruses West Nile Virus, Zika Virus, and Dengue Virus. Front. Microbiol..

[73] Wu Y.H. (2016). Naturally derived anti-hepatitis B virus agents and their mechanism of action. World J. Gastroenterol..

[74] Lim H.J., Nguyen T.T., Kim N.M., Park J.S., Jang T.S., Kim D. (2017). Inhibitory effect of flavonoids against NS2B-NS3 protease of ZIKA virus and their structure activity relationship. Biotechnol. Lett..

[75] Roy A., Lim L., Srivastava S., Lu Y., Song J. (2017). Solution conformations of Zika NS2B-NS3pro and its inhibition by natural products from edible plants. PLoS ONE.

[76] Li Z., Brecher M., Deng Y.Q., Zhang J., Sakamuru S., Liu B., Huang R., Koetzner C.A., Allen C.A., Jones S.A. (2017). Existing drugs as broad-spectrum and potent inhibitors for Zika virus by targeting NS2B-NS3 interaction. Cell Res..

[77] Yuan S., Chan J.F., den-Haan H., Chik K.K., Zhang A.J., Chan C.C., Poon V.K., Yip C.C., Mak W.W., Zhu Z. (2017). Structure-based discovery of clinically approved drugs as Zika virus NS2B-NS3 protease inhibitors that potently inhibit Zika virus infection in vitro and in vivo. Antivir. Res..

[78] Aguilar J.S., Rice M., Wagner E.K. (1999). The polysulfonated compound suramin blocks adsorption and lateral difusion of herpes simplex virus type-1 in vero cells. Virology.

[79] Baba M., Konno K., Shigeta S., Wickramasinghe A., Mohan P. (1993). Selective inhibition of human cytomegalovirus replication by naphthalenedisulfonic acid derivatives. Antivir. Res..

[80] Ellenbecker M., Lanchy J.M., Lodmell J.S. (2014). Inhibition of Rift Valley fever virus replication and perturbation of nucleocapsid-RNA interactions by suramin. Antimicrob. Agents Chemother..

[81] Wang Y., Qing J., Sun Y., Rao Z. (2014). Suramin inhibits EV71 infection. Antivir. Res..

[82] Kuo S.C., Wang Y.M., Ho Y.J., Chang T.Y., Lai Z.Z., Tsui P.Y., Wu T.Y., Lin C.C. (2016). Suramin treatment reduces chikungunya pathogenesis in mice. Antivir. Res..

[83] Ren P., Zou G., Bailly B., Xu S., Zeng M., Chen X., Shen L., Zhang Y., Guillon P., Arenzana-Seisdedos F. (2014). The approved pediatric drug suramin identified as a clinical candidate for the treatment of EV71 infection-suramin inhibits EV71 infection in vitro and in vivo. Emerg. Microbes Infect..

[84] Basavannacharya C., Vasudevan S.G. (2014). Suramin inhibits helicase activity of NS3 protein of dengue virus in a fluorescence-based high throughput assay format. Biochem. Biophys. Res. Commun..

[85] Albulescu I.C., Kovacikova K., Tas A., Snijder E.J., van Hemert M.J. (2017). Suramin inhibits Zika virus replication by interfering with virus attachment and release of infectious particles. Antivir. Res..

[86] Bekerman E., Einav S. (2015). Infectious disease. Combating emerging viral threats. Science.

[87] Graci J.D., Cameron C.E. (2006). Mechanisms of action of ribavirin against distinct viruses. Rev. Med. Virol..

[88] Sidwell R.W., Huffman J.H., Khare G.P., Allen L.B., Witkowski J.T., Robins R.K. (1972). Broad-spectrum antiviral activity of Virazole: 1-beta-D-ribofuranosyl-1,2,4-triazole-3-carboxamide. Science.

[89] Kamiyama N., Soma R., Hidano S., Watanabe K., Umekita H., Fukuda C., Noguchi K., Gendo Y., Ozaki T., Sonoda A. (2017). Ribavirin inhibits Zika virus (ZIKV) replication in vitro and suppresses viremia in ZIKV-infected STAT1-deficient mice. Antivir. Res..

[90] Tong X., Smith J., Bukreyeva N., Koma T., Manning J.T., Kalkeri R., Kwong A.D., Paessler S. (2018). Merimepodib, an IMPDH inhibitor, suppresses replication of Zika virus and other emerging viral pathogens. Antivir. Res..

[91] Rausch K., Hackett B.A., Weinbren N.L., Reeder S.M., Sadovsky Y., Hunter C.A., Schultz D.C., Coyne C.B., Cherry S. (2017). Screening Bioactives Reveals Nanchangmycin as a Broad Spectrum Antiviral Active against Zika Virus. Cell Rep..

[92] Barrows N.J., Campos R.K., Powell S.T., Prasanth K.R., Schott-Lerner G., Soto-Acosta R., Galarza-Munoz G., McGrath E.L., Urrabaz-Garza R., Gao J. (2016). A Screen of FDA-Approved Drugs for Inhibitors of Zika Virus Infection. Cell Host Microbe.

[93] Pascoalino B.S., Courtemanche G., Cordeiro M.T., Gil L.H., Freitas-Junior L. (2016). Zika antiviral chemotherapy: Identification of drugs and promising starting points for drug discovery from an FDA-approved library. F1000Research.

[94] Ikeda M., Abe K., Yamada M., Dansako H., Naka K., Kato N. (2006). Different anti-HCV profiles of statins and their potential for combination therapy with interferon. Hepatology.

[95] Rothwell C., Lebreton A., Young Ng C., Lim J.Y., Liu W., Vasudevan S., Labow M., Gu F., Gaither L.A. (2009). Cholesterol biosynthesis modulation regulates dengue viral replication. Virology.

[96] Sarkey J.P., Richards M.P., Stubbs E.B. (2007). Lovastatin attenuates nerve injury in an animal model of Guillain-Barre syndrome. J. Neurochem..

[97] Retallack H., Di Lullo E., Arias C., Knopp K.A., Laurie M.T., Sandoval-Espinosa C., Mancia Leon W.R., Krencik R., Ullian E.M., Spatazza J. (2016). Zika virus cell tropism in the developing human brain and inhibition by azithromycin. Proc. Natl. Acad. Sci. USA.

[98] Lin K.J., Mitchell A.A., Yau W.P., Louik C., Hernandez-Diaz S. (2013). Safety of macrolides during pregnancy. Am. J. Obstet Gynecol..

[99] Al-Bari M.A.A. (2017). Targeting endosomal acidification by chloroquine analogs as a promising strategy for the treatment of emerging viral diseases. Pharmacol. Res. Perspect..

[100] Levy M., Buskila D., Gladman D.D., Urowitz M.B., Koren G. (1991). Pregnancy outcome following first trimester exposure to chloroquine. Am. J. Perinatol..

[101] Delvecchio R., Higa L.M., Pezzuto P., Valadao A.L., Garcez P.P., Monteiro F.L., Loiola E.C., Dias A.A., Silva F.J., Aliota M.T. (2016). Chloroquine, an Endocytosis Blocking Agent, Inhibits Zika Virus Infection in Different Cell Models. Viruses.

[102] Miner J.J., Cao B., Govero J., Smith A.M., Fernandez E., Cabrera O.H., Garber C., Noll M., Klein R.S., Noguchi K.K. (2016). Zika Virus Infection during Pregnancy in Mice Causes Placental Damage and Fetal Demise. Cell.

[103] Kuivanen S., Bespalov M.M., Nandania J., Ianevski A., Velagapudi V., De Brabander J.K., Kainov D.E., Vapalahti O. (2017). Obatoclax, saliphenylhalamide and gemcitabine inhibit Zika virus infection in vitro and differentially affect cellular signaling, transcription and metabolism. Antivir. Res..

[104] Jurgeit A., McDowell R., Moese S., Meldrum E., Schwendener R., Greber U.F. (2012). Niclosamide is a proton carrier and targets acidic endosomes with broad antiviral effects. PLoS Pathog..

[105] Xu M., Lee E.M., Wen Z., Cheng Y., Huang W.K., Qian X., Tcw J., Kouznetsova J., Ogden S.C., Hammack C. (2016). Identification of small-molecule inhibitors of Zika virus infection and induced neural cell death via a drug repurposing screen. Nat. Med..

[106] Costa V.V., Del Sarto J.L., Rocha R.F., Silva F.R., Doria J.G., Olmo I.G., Marques R.E., Queiroz-Junior C.M., Foureaux G., Araujo J.M.S. (2017). N-Methyl-d-Aspartate (NMDA) Receptor Blockade Prevents Neuronal Death Induced by Zika Virus Infection. mBio.

